# Antibiotic Management Following Urine and Blood Culture Results: A Retrospective Single-Centre Observational Study at University Hospital Hairmyres, United Kingdom

**DOI:** 10.7759/cureus.115477

**Published:** 2026-08-31

**Authors:** Yoosuf Y Ibrahim

**Affiliations:** 1 Geriatrics, University Hospital Hairmyres, East Kilbride, GBR

**Keywords:** antibiotic resistance, antibiotic stewardship, blood culture, clinical audit, urinary tract infection, urine culture

## Abstract

Background

Timely review and actioning of microbiological culture results are essential for effective antimicrobial stewardship. Delays in modifying therapy when cultures demonstrate resistance may result in prolonged exposure to ineffective antimicrobials and suboptimal management. This study aimed to evaluate the timeliness and appropriateness of antibiotic initiation and modification following positive urine and blood culture results in a district general hospital and to compare management between these two specimen types.

Methods

A retrospective observational study was conducted at University Hospital Hairmyres, United Kingdom, analysing 100 positive urine cultures and 25 positive blood cultures collected between the 4th and 17th December 2023. Patients with repeat samples or *Candida *infections were excluded. Data were extracted from the NHS clinical portal and the Hospital Electronic Prescribing and Medicines Administration (HEPMA) system. Variables included empirical antibiotic use, antimicrobial sensitivities, and time to initiation or modification of therapy following availability of culture and sensitivity results. Thirteen predefined study questions were used to assess antimicrobial management. "Appropriate modification" was defined as changing to an agent to which the organism was reported susceptible, taking into account route and documented allergies where available; clinical factors, such as renal function, infection site, and local guidance, were not systematically captured.

Results

A total of 125 patients were included (mean age: 71.9 years, SD: 16.8; 64% female). Among urine culture patients, 76/100 (76.0%) received antibiotics, including 59/100 (59.0%) prescribed empirical therapy. Of those receiving empirical treatment with susceptibility data available for the prescribed drug (n = 36), 15/36 (41.7%) received an antibiotic to which the organism was resistant. In this resistant group, 6/15 (40.0%) were not switched to a susceptible agent despite available results. Where therapy was modified appropriately, the mean delay between availability of sensitivity results and modification was 2.22 days. Among 41 patients not initially prescribed empirical antibiotics, 17 were commenced on treatment following culture positivity, with a mean delay of 2.24 days. All 25 blood culture patients received antibiotics, including 24/25 (96.0%) prescribed empirical therapy. Resistant empirical therapy was identified in 2/24 (8.3%) cases; both were appropriately changed to a susceptible agent prior to formal sensitivity reporting. Resistant empirical therapy was more frequent in urine than blood culture episodes when analysis was restricted to cases with interpretable susceptibility data (15/36 (41.7%) vs 2/24 (8.3%), Fisher's exact test, p = 0.0075). Empirical therapy was significantly more common in blood than urine cultures (24/25 (96.0%) vs 59/100 (59.0%), p=0.001).

Conclusions

This study identified delays in modifying antibiotic therapy following positive urine culture results, with a substantial proportion of patients remaining on resistant empirical regimens despite available sensitivity data. Findings suggest gaps in the timely review and optimisation of therapy, particularly for urine cultures. Implementation of enhanced notification systems and structured review processes may support more timely antimicrobial decision‑making and strengthen stewardship.

## Introduction

Urinary tract infections (UTIs) represent a significant burden within hospital settings and are among the most common healthcare-associated infections. Despite their frequency, there is considerable variability in how urine cultures are ordered, interpreted, and acted upon in routine practice. A large multicentre study published in the New England Journal of Medicine, involving patients across 183 hospitals, identified UTIs as a major contributor to healthcare-associated morbidity and associated costs [1].

A key challenge is the lack of awareness among healthcare providers regarding newly available urine culture results. This can lead to delays in adjusting antimicrobial therapy, particularly when the initial empirical regimen is ineffective due to resistance. Such delays may adversely impact patient outcomes and contribute to the propagation of antimicrobial resistance. A study conducted in Jordan involving 2,400 positive urine cultures highlighted the clinical importance of timely response to microbiological data, demonstrating that delayed modification of antibiotics in the presence of resistance is associated with increased mortality in patients with bloodstream infections [2]. Furthermore, the study revealed a high incidence of inappropriate UTI management despite the availability of culture and sensitivity data, suggesting underutilisation of microbiological information in guiding treatment decisions [2].

Continuation of ineffective antibiotics due to delayed response to culture results poses substantial risks to patient safety. Resistance to commonly prescribed UTI therapies, including fluoroquinolones and trimethoprim, is an increasing global concern [3]. Prolonged use of ineffective antimicrobial therapy not only fails to adequately treat infection but also promotes the emergence and dissemination of multidrug-resistant organisms, thereby complicating future treatment strategies [3]. In contrast, blood cultures are generally ordered and actively reviewed by physicians, resulting in higher responsiveness to microbiological findings. This increased clinical engagement is associated with improved adherence to treatment guidelines and more timely optimisation of antimicrobial therapy in bloodstream infections. Physician involvement in both the ordering and interpretation of blood cultures is therefore critical for ensuring prompt and appropriate clinical decision-making [4].

This study was conducted to evaluate current practice in the management of positive urine and blood cultures at a district general hospital. The primary objectives were to assess the timeliness and appropriateness of antibiotic initiation and modification following positive urine and blood culture results, compare management between urine and blood culture episodes, and identify system‑level factors contributing to delays in therapy optimisation. Comparing urine and blood cultures allowed assessment of differences in clinical urgency, review processes, and responsiveness to microbiology results between these common specimen types.

## Materials and methods

This study was conducted as a retrospective single-centre observational study at University Hospital Hairmyres, United Kingdom, in January 2024, evaluating the management of patients with positive urine and blood culture results. The study assessed antimicrobial prescribing and review processes following availability of microbiology results.

Setting and sample

Data were collected for 100 consecutive positive urine cultures and 25 consecutive positive blood cultures processed between 4 and 17 December 2023 in all areas of the hospital. Inclusion criteria were adult patients with a positive bacterial urine or blood culture during the study period. Exclusion criteria were repeat cultures from the same patient within the study period and cultures yielding *Candida *species.

Data sources and collection

Data were extracted from the NHS clinical portal and the Hospital Electronic Prescribing and Medicines Administration (HEPMA) system. Microbiology results were considered "available" from the time the final culture, and sensitivity report were issued in the laboratory information system and visible in the clinical portal. In our institution, new results are highlighted in bold within the portal when first released, but they are not prominently displayed on the main ward screens and may not be apparent at first glance without actively opening the microbiology tab.

Variables collected included patient age and sex, clinical location/ward, empirical antibiotic prescribing, antimicrobial sensitivity profiles, timing of antibiotic initiation, and any subsequent modifications to therapy following availability of microbiology results.

Study questions and definitions

Thirteen predefined study questions were developed to assess antimicrobial management following microbiological culture results (Table 1). These questions evaluated empirical prescribing, concordance of therapy with susceptibility results, modification of therapy when resistance was identified, and timeliness of initiation or escalation.

**Table 1:** Predefined study questions used to assess antimicrobial management

Study parameter	Study question	Purpose
1	Was antibiotic therapy prescribed following a positive urine culture?	Assess overall antibiotic prescribing
2	Was empirical antibiotic therapy initiated before urine culture results were available?	Assess empirical prescribing practice
3	Was the empirical antibiotic active against the cultured organism in the urine?	Assess concordance with susceptibility
4	If resistant, was therapy changed appropriately?	Assess response to microbiology results
5	What was the delay between availability of susceptibility results and antibiotic modification?	Assess timeliness of escalation
6	If no empirical therapy was given, was treatment started after culture positivity?	Assess delayed initiation
7	What was the delay between culture positivity and antibiotic initiation?	Assess timeliness of treatment
8	Were there cases where susceptibility testing was unavailable or not reported?	Identify limitations affecting prescribing
9	Which clinical areas/wards were associated with failure to modify resistant therapy?	Identify potential targets for quality improvement
10	Were blood culture isolates clinically significant?	Assess interpretation of blood culture results
11	Was empirical blood culture therapy concordant with sensitivities?	Assess appropriateness of blood culture management
12	If resistant, was blood culture therapy modified appropriately?	Assess response to blood culture results
13	What was the delay between blood culture reporting and treatment escalation?	Assess timeliness of blood culture management

The following terms were defined for this study: (1) "Resistant empirical therapy" was defined as an empirical antibiotic to which the cultured organism was reported resistant on the final sensitivity profile. (2) "Appropriate modification" was defined as changing the antibiotic regimen to an agent to which the organism was reported susceptible, with a suitable route, and with no documented contraindication (e.g., allergy) in the prescribing record. Clinical factors, such as renal function, infection site, drug interactions, and local guideline concordance, were not systematically captured and therefore not included in this definition. (3) "Delay to modification" was calculated as the number of days between the date and time the final sensitivity result became available in the clinical portal and the date and time the antibiotic order was changed in HEPMA. Same‑day changes were recorded as 0 days. (4) "Empirical therapy" referred to antibiotics prescribed before the final culture and sensitivity result was available. Separate but equivalent questions were applied to urine and blood culture episodes.

Analysis

Data were analysed descriptively. Proportions were calculated with clearly defined denominators. For resistance analyses, we report both (a) resistance among all patients receiving empirical therapy and (b) resistance among patients for whom susceptibility to the prescribed drug could be assessed. Fisher's exact test was used to compare proportions between urine and blood culture groups where appropriate. No formal statistical power calculation was performed; the sample comprised all eligible cultures within the two‑week period.

Ethics

As a service evaluation undertaken to assess local practice, this project did not require formal research ethics committee approval. Patient data were handled in accordance with local governance and data protection requirements.

## Results

A total of 125 patients were included: 100 with positive urine cultures and 25 with positive blood cultures. The mean age was 71.9 years (SD: 16.8), with a median of 75 years (IQR: 65-84) and a range of 20-101 years. Females accounted for 80/125 (64.0%) and males for 45/125 (36.0%). The most represented age groups were 80-89 years (39, 31.2%) and 70-79 years (37, 29.6%), together comprising 76/125 (60.8%). Detailed demographic characteristics are presented in Table 2. 

**Table 2:** Demographics

Age	
Age Group (years)	N (%)
20-29	4 (3.2)
30-39	5 (4.0)
40-49	3 (2.4)
50-59	11 (8.8)
60-69	18 (14.4)
70-79	37 (29.6)
80-89	39 (31.2)
90-99	7 (5.6)
≥100	1 (0.8)
Gender	
Gender	N (%)
Male	45 (36.0)
Female	80 (64.0)

Urine culture findings

Among 100 positive urine cultures, 76 (76.0%) patients received antibiotic therapy either before or after culture results. Of these, 59 (59.0% of all urine culture patients) were prescribed empirical antibiotics prior to availability of sensitivity results.

Of the 59 patients receiving empirical therapy, susceptibility data for the prescribed drug were available in 36 cases. In the remaining 23 cases, the prescribed antibiotic was not included in the sensitivity panel, no sensitivity result was reported, or testing was not performed. Among the 36 episodes with interpretable susceptibility data, 21 (58.3%) received an antibiotic to which the organism was susceptible, and 15 (41.7%) received an antibiotic to which the organism was resistant.

Among the 15 patients receiving resistant empirical therapy, treatment was modified to a susceptible agent in nine cases (60.0%). In six cases (40.0%), no change in therapy occurred despite available microbiology results.

In episodes where therapy was appropriately modified, the interval between availability of sensitivity results and modification ranged from 0 to 7 days, with a mean delay of 2.22 days. Two patients (22.2%) had same‑day modification, while one (11.1%) experienced a seven‑day delay (Table 3).

**Table 3:** Delay in modification of antibiotic therapy following resistant urine culture results

Days to modification	n (%)
0	2 (22.2)
1	2 (22.2)
2	2 (22.2)
3	1 (11.1)
4	1 (11.1)
7	1 (11.1)
Mean delay	2.22 days

Among patients not initially prescribed empirical antibiotics (n = 41), 17 were started on treatment following a positive culture. Time to initiation ranged from 0 to 14 days, with a mean delay of 2.24 days. Nine patients (52.9%) commenced antibiotics on the same day results became available.

Location‑based analysis showed that the emergency department accounted for the highest proportion of cases in which resistant antibiotics for urine cultures were not adjusted, representing 3/6 (50.0%) of such cases.

Of the 59 urine culture patients receiving empirical therapy, susceptibility data for at least one prescribed empirical antibiotic were available in 36 episodes. In these episodes, a total of 39 empirical antibiotic orders were recorded because some patients received more than one agent. Among the common empirical antibiotics used for urine cultures, nitrofurantoin showed the highest susceptibility rate, with 7/7 (100.0%) isolates sensitive and none resistant. Trimethoprim was sensitive in 8/11 (72.7%) and resistant in 3/11 (27.3%) cases. Fosfomycin was sensitive in 5/6 (83.3%) and resistant in 1/6 (16.7%) cases. In contrast, amoxicillin was sensitive in 4/11 (36.4%) and resistant in 7/11 (63.6%) cases, while co-amoxiclav showed the lowest susceptibility, with 1/4 (25.0%) sensitive and 3/4 (75.0%) resistant.

Notable individual findings included two cases of *Enterococcus faecalis *in which trimethoprim sensitivity testing was not performed; in both cases, patients continued to receive trimethoprim without subsequent modification. One case demonstrated a prolonged delay of seven days before escalation from a resistant to a susceptible regimen. 

Blood culture findings

All 25 patients with positive blood cultures received antibiotic therapy, with 24 (96.0%) already on empirical treatment prior to availability of culture results.

Sensitivity analysis showed that 10 patients (40.0%) received antibiotics to which the organism was susceptible, while two patients (8.3%) received antibiotics to which the organism was resistant. Nine cultures (36.0%) were deemed clinically insignificant by the treating team. In addition, three patients (12.0%) were prescribed antibiotics not included in the sensitivity panel, and one case (4.0%) had no sensitivity testing performed.

In both cases involving resistant empirical therapy, treatment was appropriately escalated to a susceptible agent prior to formal reporting of sensitivity results (Table 4). As such, no measurable delay in response to sensitivity data was identified.

**Table 4:** Comparison of antimicrobial management following urine and blood culture results For resistant empirical antibiotics, susceptibility data for the prescribed empirical antibiotic were interpretable in 36/59 urine culture episodes and 24/24 blood culture episodes. The comparison of resistance rates was therefore based on these interpretable episodes (15/36 vs 2/24).

Outcome	Urine cultures (n=100)	Blood cultures (n=25)	Statistical test	p-value
Empirical antibiotics prescribed	59 (59.0%)	24 (96.0%)	Fisher's exact test	0.001
Resistant empirical antibiotics among episodes with interpretable susceptibility data	15/36 (41.7%)	2/24 (8.3%)	Fisher's exact test	0.0075
Resistant antibiotics appropriately changed	9/15 (60.0%)	2/2 (100.0%)	Fisher's exact test	0.515
Resistant antibiotics not changed	6/15 (40.0%)	0	Fisher's exact test	Not calculated due to zero events in blood culture group
Mean delay to modification (days)	2.22	0	Descriptive	-

A notable case involved methicillin‑resistant *Staphylococcus aureus *(MRSA), where microbiology results were communicated by telephone to a consultant no longer covering the ward. Vancomycin therapy was initiated one day later, while the interim culture result was not formally reported until 16 days after sample collection. Another case of *Corynebacterium *species did not undergo sensitivity testing despite ongoing treatment with amoxicillin and co‑amoxiclav.

Comparative analysis

Comparative analysis demonstrated that resistant empirical antibiotic therapy was more common in patients with positive urine cultures than in those with positive blood cultures when analysis was restricted to episodes with interpretable susceptibility data (15/36 (41.7%) vs 2/24 (8.3%), Fisher's exact test, p = 0.0075). The proportion of resistant empirical antibiotics appropriately modified did not differ significantly between urine and blood cultures (9/15 (60.0%) vs 2/2 (100.0%), p=0.515). Empirical therapy was significantly more common in blood than urine cultures (24/25 (96.0%) vs 59/100 (59.0%), p=0.001), reflecting greater clinical urgency associated with suspected bloodstream infection (Table 4).

## Discussion

This study identified important gaps in the timely optimisation of antibiotic therapy following positive microbiology results, particularly for urine cultures. Most notably, among urine culture patients receiving empirical therapy with interpretable susceptibility data, 41.7% were prescribed an antibiotic to which the organism was resistant, and 40.0% of these resistant regimens were not changed despite available results. This indicates that availability of microbiological information did not consistently result in antimicrobial optimisation. Possible contributing factors include passive review processes, competing clinical priorities, or uncertainty about the clinical significance of positive urine cultures [5,6].

Although therapy was appropriately modified in 60.0% of resistant urine culture episodes, the mean delay of 2.22 days between result availability and treatment adjustment represents a potentially avoidable period of ineffective therapy. Timely review and optimisation of antibiotics are fundamental to antimicrobial stewardship, enabling escalation when empirical therapy is inadequate and de‑escalation when narrower‑spectrum options are appropriate. Prior studies have shown that inappropriate or prolonged antimicrobial exposure increases selection pressure for resistant organisms and that stewardship interventions can improve prescribing quality [6,7].

Interpretation of urine cultures requires particular caution, as microbiological positivity does not always indicate clinically significant infection. Positive urine cultures may reflect asymptomatic bacteriuria, colonisation, or contamination, especially in older or catheterised patients. Infectious Diseases Society of America (IDSA) guidelines emphasise that urine culture results should be interpreted alongside clinical symptoms and patient factors rather than used as the sole determinant for treatment [8]. In this study, we did not systematically capture symptom data or clinical indications for treatment, so some instances of non‑modification may have been clinically appropriate (e.g., treatment of asymptomatic bacteriuria not indicated). This is an important limitation.

A contributing factor to delayed or absent modification may be the lack of a standardised, proactive process for reviewing clinically significant microbiology results. In our institution, new results are highlighted in bold within the clinical portal when first released, but they are not prominently displayed on main ward screens and may not be apparent without actively opening the microbiology tab. Reliance on passive review of electronic systems may therefore introduce variability and increase the risk of delayed intervention. Previous studies have demonstrated that rapid communication of microbiological findings, particularly when combined with stewardship input, can improve the timeliness and appropriateness of therapy [9,10]. Electronic alerts, clinical decision‑support tools, and structured notification pathways may therefore be valuable, provided that they are supported by clear responsibility for result review and effective collaboration between microbiology, pharmacy, and clinical teams.

Behavioural and organisational factors also influence stewardship. Improving awareness of the importance of reviewing microbiology results and reinforcing expectations around antimicrobial reassessment may improve consistency. Multifaceted stewardship interventions incorporating education, review and feedback, prospective review, and clinical support are more effective than isolated educational approaches [5,11]. Structured antimicrobial review processes, such as mandatory review of positive cultures within a defined timeframe, could be a practical next step locally.

Findings relating to blood cultures should be interpreted cautiously due to the small sample size and limited number of resistant cases. The high proportion of patients receiving empirical antibiotics likely reflects the clinical severity and urgency of suspected bloodstream infection. Both resistant blood culture episodes were appropriately escalated before formal susceptibility reporting, limiting assessment of delayed modification. However, individual cases, including delayed communication of MRSA results, highlight the importance of effective communication pathways for clinically significant bloodstream infections [9,10].

The susceptibility patterns of commonly used empirical urinary antibiotics in this study also underscore the importance of aligning prescribing choices with local resistance data. Nitrofurantoin and fosfomycin demonstrated high in vitro activity, whereas amoxicillin and co‑amoxiclav showed substantially higher resistance rates. These findings suggest that empirical use of broad-spectrum agents, such as co‑amoxiclav, may not confer a sensitivity advantage over narrower-spectrum options, and highlight the value of regularly reviewing local antibiograms when selecting empirical therapy for suspected UTI.

Limitations

This study has several limitations. As a single‑centre retrospective study, findings may not be generalisable to other institutions with different microbiology reporting systems or stewardship structures. The sample size, particularly for blood cultures and resistant blood culture episodes, was small, limiting statistical power. Retrospective assessment relied on available documentation; undocumented clinical reasoning (e.g., decision not to treat asymptomatic bacteriuria) could not be captured. We did not systematically record organism types, resistance patterns by drug class, clinical locations beyond ward level, or the full range of empirical regimens prescribed, which limits the depth of insight into prescribing patterns. Finally, our definition of "appropriate modification" was based primarily on susceptibility and route, without systematic assessment of renal function, infection site, drug interactions, or local guideline concordance.

Despite these limitations, this study provides real‑world insight into antimicrobial management following microbiological reporting. The findings suggest that availability of culture and sensitivity results alone does not ensure timely optimisation. Effective stewardship requires systems that ensure results are recognised, communicated, interpreted, and acted upon appropriately.

Next steps

Based on these findings, planned or recommended next steps include local discussion with microbiology and pharmacy teams regarding enhanced notification of clinically significant results, consideration of a structured review process for positive cultures (e.g., review within 24-48 hours of result availability), targeted education for teams in areas with frequent delays (e.g., emergency department), and re‑review after implementation of any interventions to assess improvement.

## Conclusions

This study identified delays in the modification of antibiotic therapy following positive urine culture results, with a substantial proportion of patients remaining on resistant empirical regimens despite available sensitivity data. These findings highlight potential deficiencies in the timely review and optimisation of microbiology results, with implications for patient safety and antimicrobial stewardship.

Implementation of enhanced notification systems and structured review processes may improve clinician awareness of newly available results and facilitate more timely antibiotic optimisation. Further studies with larger sample sizes, detailed organism and drug data, and inclusion of clinical indications and patient outcomes are warranted to better evaluate the impact of these delays and the effectiveness of quality improvement interventions.
